# How central is dopamine to pathological gambling or gambling disorder?

**DOI:** 10.3389/fnbeh.2013.00206

**Published:** 2013-12-23

**Authors:** Marc N. Potenza

**Affiliations:** Departments of Psychiatry, Neurobiology, and Child Study Center, Yale University School of MedicineNew Haven, CT, USA

**Keywords:** dopamine, gambling, addiction, PET, serotonin, glutamate, opioids

Pathological gambling [PG—now termed “gambling disorder” in DSM-5 (APA, 2013; Petry et al., 2013)] is characterized by maladaptive patterns of gambling that are associated with significant impairments in functioning. Over the past decade, significant advances have been made in understanding the pathophysiology of PG (Potenza, 2013). Similarities between PG and substance-use disorders (Petry, 2006; Potenza, 2006; Leeman and Potenza, 2012) prompted the reclassification of PG in DSM-5 as an addictive disorder (rather than an impulse-control disorder, as was the case in DSM-IV).

Multiple neurotransmitter systems have been implicated in PG including serotonergic, noradrenergic, dopaminergic, opioidergic, and glutamatergic (Potenza, 2013). An understanding of these systems as they relate to PG is important clinically for drug development as presently there are no FDA-approved medications with indications for PG. Dopamine has long been implicated in substance addictions and early articles postulated a similarly important role for dopamine in PG (Potenza, 2001). However, a precise role for dopamine in PG remains unclear. Studies of cerebrospinal fluid samples indicated low levels of dopamine and high levels of dopamine metabolites in PG, raising the possibility of increased dopamine turnover (Bergh et al., 1997). However, medications that target dopamine function have not demonstrated clinical effects in PG. For example, medications that block dopamine D2-like receptor function (e.g., olanzapine) have shown negative results in small, randomized clinical trials (Fong et al., 2008; McElroy et al., 2008). Furthermore, a D2-like dopamine receptor antagonist widely used in the treatment of psychotic disorders (haloperidol) was found to increase gambling-related motivations and behaviors in individuals with PG (Zack and Poulos, 2007). However, administration of the pro-dopaminergic (and pro-adrenergic) drug amphetamine also led to increased gambling-related thoughts and behaviors in PG (Zack and Poulos, 2004).

Recent imaging studies have begun to use radioligands and positron-emission tomography to investigate dopamine function in PG. In contrast to findings in cocaine dependence in which between-group differences were observed in [^11^C]raclopride-binding in the striatum, similar levels were observed in PG and comparison subjects by two investigative groups (Linnet et al., 2010, 2011; Clark et al., 2012). Similarly, no between-group difference between PG and comparison subjects was observed using [^11^C]raclopride or the D3-preferring agonist-radioligand [^11^C]-(+)-propyl-hexahydro-naphtho-oxazin (PHNO) (Boileau et al., 2013). However, in these studies, relationships with mood-related or generalized impulsivity, disadvantageous decision-making or problem-gambling severity were reported, suggesting that dopamine function may relate to specific aspects of PG (Potenza and Brody, 2013). These findings are consistent with the idea that PG represents a heterogeneous condition and that identifying biologically relevant individual differences or subgroups may help advance treatment development or the appropriate targeting of therapeutic interventions.

A now well-documented association between dopamine and PG exists in Parkinson's disease (PD) (Leeman and Potenza, 2011). Specifically, dopamine agonists (e.g., pramipexole, ropinirole) have been associated with PG and excessive or problematic behaviors in other domains (relating to sex, eating, and shopping) in individuals with PD (Weintraub et al., 2010). Furthermore, levodopa dosing has also been associated with these conditions in PD (Weintraub et al., 2010). However, factors seemingly unrelated to dopamine (e.g., age of PD onset, marital status and geographic location) have also been associated with these conditions in PD (Voon et al., 2006; Weintraub et al., 2006, 2010; Potenza et al., 2007), highlighting the complicated etiologies of these disorders. Nonetheless, in a study using [^11^C]raclopride, individuals with PD and PG as compared to those with PD alone demonstrated in the ventral (but not dorsal) striatum diminished D2-like binding at baseline and greater [^11^C]raclopride displacement during a gambling/decision-making task (suggesting greater dopamine release in the PG group during task performance) (Steeves et al., 2009). These findings are reminiscent of those suggesting blunted levodopa-induced displacement of [^11^C]raclopride in the ventral but not dorsal striatum in PD subjects who self-administer dopamine-replacement therapies to excess (as compared to those who do not) (Evans et al., 2006). As other findings have identified in association with behavioral addictions in PD (vs. those with PD alone) relatively reduced signal in the ventral striatum at baseline and during risk-taking (Rao et al., 2010), a question arises as to whether dopamine might relate to these processes in PD. Similar questions exist about the relatively blunted ventral striatal activation seen in non-PD PG in non-ligand-based imaging during simulated gambling (Reuter et al., 2005) and monetary reward processing (Balodis et al., 2012a; Choi et al., 2012). Although multiple studies have found blunted ventral striatal activation during the monetary-reward-anticipation phase (particularly during the performance of Monetary Incentive Delay tasks) across multiple addictive disorders [e.g., alcohol-use (Wrase et al., 2007; Beck et al., 2009) and tobacco-use (Peters et al., 2011) disorders] and other conditions characterized by impaired impulse control [e.g., binge-eating disorder (Balodis et al., 2013, in press)], other studies have found relatively increased ventral striatal activation during reward processing in individuals with PG and those with other addictions (Hommer et al., 2011; van Holst et al., 2012a), further raising questions about how striatal function contributes precisely to PG and addictions and how dopamine may be involved in these processes (Balodis et al., 2012b; Leyton and Vezina, 2012; van Holst et al., 2012b).

Although much of the radioligand-related data described above investigate D2/D3 receptor function, other dopamine receptors warrant consideration in PG. For example, on a rodent slot-machine task, the D2-like receptor agonist quinpirole enhanced erroneous expectations of reward on near-miss trials, and this effect was attenuated by a selective D4 (but not D3 or D2) dopamine receptor antagonist (Cocker et al., 2013). These preclinical findings complement human studies that suggest a role for the D4 dopamine receptor in gambling behaviors. For example, allelic variation at the gene coding for the D4 dopamine receptor has been associated with differential responses to levodopa-related increases in gambling behaviors (Eisenegger et al., 2010). These findings complement a larger literature linking the D4 dopamine receptor to impulsivity-related constructs and disorders like attention-deficit/hyperactivity disorder, albeit somewhat inconsistently (Ebstein et al., 1996; Gelernter et al., 1997; DiMaio et al., 2003). As preclinical (Fairbanks et al., 2012) and human (Sheese et al., 2012) data suggest gene-by-environment interactions involving the gene encoding the D4 dopamine receptor and aspects of impulsive or poorly controlled behaviors, further research should examine a role for the D4 dopamine receptor in PG, particularly in studies employing careful assessments of environmental and genetic factors. Although several D4-preferring/selective agonist compounds (e.g., PD-168,077 and CP-226,269) have been used in preclinical studies to study D4 receptors, additional research is needed to study human D4 dopamine receptors as might be accomplished through positron-emission-tomography studies—this represents an important line of future research (Bernaerts and Tirelli, 2003; Tarazi et al., 2004; Basso et al., 2005). Additionally, as the D1 dopamine receptor has been implicated in addictions like cocaine dependence (Martinez et al., 2009), a role for the D1 dopaminergic system in PG warrants exploration.

The above findings indicate that how dopaminergic function may contribute to PG and other addictions is currently at an early stage of understanding. Current data suggest that individual variability in dopamine function may obscure differences between PG and non-PG populations, with arguably the strongest between-group differences to date observed in a group with dopaminergic pathology (PD). The individual characteristics (e.g., impulsivity, decision-making and gambling-related behaviors) linked to dopamine function in PG and non-PG subjects also warrant consideration from a clinical perspective and suggest that these might represent novel treatment targets that link particularly closely to biological function [raising the possibility that they may be particularly amenable to targeting with medications (Berlin et al., 2013)]. Additionally, other potential endophenotypes like compulsivity (Fineberg et al., 2010, in press) warrant consideration given their preliminary links to treatment outcome in PG (Grant et al., 2010). Additionally, systems that may regulate dopamine function warrant further consideration in treatment development. For example, in randomized clinical trials, opioid antagonists like nalmefene and naltrexone have been found to be superior to placebo in treating PG (Grant et al., 2006, 2008b), particularly amongst individuals with strong gambling urges or familial histories of alcoholism (Grant et al., 2008a). Similarly, glutamatergic systems warrant consideration in this regard (Kalivas and Volkow, 2005), with preliminary data linking the neutraceutical n-acetyl cysteine to positive treatment outcome in PG (Grant et al., 2007). As dissecting the dopamine system is providing insight into PG, similar approaches should be used to investigate serotonin function in PG (Potenza et al., 2013), particularly given inconsistent findings with serotonergic medications in the treatment of PG (Bullock and Potenza, 2012). A systematic approach to investigating the neurobiology and clinical characteristics of PG should help advance prevention and treatment strategies for PG.

## Disclosures

Dr. Marc N. Potenza has no financial conflicts of interest with respect to the content of this manuscript and has received financial support or compensation for the following: Dr. Marc N. Potenza has consulted for and advised Boehringer Ingelheim, Ironwood, and Lundbeck; has consulted for and has financial interests in Somaxon; has received research support from Mohegan Sun Casino, the National Center for Responsible Gaming, Forest Laboratories, Ortho-McNeil, Oy-Control/Biotie, Psyadon, Glaxo-SmithKline, the National Institutes of Health and Veteran's Administration; has participated in surveys, mailings or telephone consultations related to drug addiction, impulse control disorders or other health topics; has consulted for law offices and the federal public defender's office in issues related to impulse control disorders; provides clinical care in the Connecticut Department of Mental Health and Addiction Services Problem Gambling Services Program; has performed grant reviews for the National Institutes of Health and other agencies; has guest-edited journal sections; has given academic lectures in grand rounds, CME events and other clinical or scientific venues; and has generated books or book chapters for publishers of mental health texts.
